# Focal Adhesion Protein Vinculin Is Required for Proper Meiotic Progression during Mouse Spermatogenesis

**DOI:** 10.3390/cells11132013

**Published:** 2022-06-23

**Authors:** Jana Petrusová, Robert Havalda, Petr Flachs, Tomáš Venit, Alžběta Darášová, Lenka Hůlková, Martin Sztacho, Pavel Hozák

**Affiliations:** 1 Department of Biology of the Cell Nucleus, Institute of Molecular Genetics of the Czech Academy of Sciences, Vídeňská 1083, 142 20 Prague, Czech Republic; jana.petrusova@img.cas.cz (J.P.); robert.havalda@gmail.com (R.H.); flachs@gymkh.eu (P.F.); tv21@nyu.edu (T.V.); alzbeta.darasova@fnkv.cz (A.D.); lenkaa.hulkova@gmail.com (L.H.); sztacho@img.cas.cz (M.S.); 2 Department of Immunobiology, Institute of Molecular Genetics of the Czech Academy of Sciences, Vídeňská 1083, 142 20 Prague, Czech Republic; 3 Science Division, Biology Program, New York University Abu Dhabi (NYUAD), Abu Dhabi P.O. Box 129188, United Arab Emirates

**Keywords:** vinculin, spermatogenesis, centromere synapsis, kinetochore, ubiquitin–proteasome system, fertility

## Abstract

The focal adhesion protein Vinculin (VCL) is ascribed to various cytoplasmic functions; however, its nuclear role has so far been ambiguous. We observed that VCL localizes to the nuclei of mouse primary spermatocytes undergoing first meiotic division. Specifically, VCL localizes along the meiosis-specific structure synaptonemal complex (SC) during prophase I and the centromeric regions, where it remains until metaphase I. To study the role of VCL in meiotic division, we prepared a conditional knock-out mouse (VCL^cKO^). We found that the VCL^cKO^ male mice were semi-fertile, with a decreased number of offspring compared to wild-type animals. This study of events in late prophase I indicated premature splitting of homologous chromosomes, accompanied by an untimely loss of SCP1. This caused erroneous kinetochore formation, followed by failure of the meiotic spindle assembly and metaphase I arrest. To assess the mechanism of VCL involvement in meiosis, we searched for its possible interacting partners. A mass spectrometry approach identified several putative interactors which belong to the ubiquitin–proteasome pathway (UPS). The depletion of VLC leads to the dysregulation of a key subunit of the proteasome complex in the meiotic nuclei and an altered nuclear SUMOylation level. Taken together, we show for the first time the presence of VCL in the nucleus of spermatocytes and its involvement in proper meiotic progress. It also suggests the direction for future studies regarding the role of VCL in spermatogenesis through regulation of UPS.

## 1. Introduction

During meiosis, sexually reproducing organisms produce gametes containing a single set of chromosomes. Meiosis consists of a DNA replication step, followed by two consecutive chromosome segregations resulting in four haploid gametes [1,2,3,4]. During the first meiotic division, homologous chromosomes (homologs) pair up, connect along their lengths on the proteinaceous structure called a synaptonemal complex (SC), recombine, and dissociate [5,6]. Successful completion of these events is critical to proper chromosome segregation. Aberrations during chromosome segregation are the major cause of genetic instability, which often leads to the progression of various malignities [7]. The tripartite SC structure, which consists of two lateral elements and central element, is required for the formation of synapsis between homologs [8,9,10]. The SC plays a key role in the recombination event called crossing over by formation of chiasmata. Chiasmata provide a physical link between homologs and ensure proper chromosome orientation and segregation during the first meiotic division [11]. Nascent chiasmata and centromeres are typically the last regions of homologs which are synapsed [12]. In mouse meiosis I, the persistent association of centromeres between homologs and sister chromatids directs proper chromosome segregation, the spindle assembly checkpoint (SAC), and attachment to microtubules (MTs) from the opposite poles [13,14,15]. The removal of Synaptonemal complex protein 1 (SCP1) initiates a disassembly of the SC, thereby defining the beginning of diplotene. The association of centromeres persists until early diakinesis, where the last residues of SCP1 localize with coupled centromeres and nascent chiasmata. By contrast, SCP3 persists and is associated with centromeres until anaphase I [12,16,17]. SCP1 phosphorylation ensures SC degradation by disrupting protein interaction during diplotene, and initiates the progression from prophase I to diplotene and to the first metaphase (G2/MI transition) [16]. Metaphase-promoting factor (MPF) and Aurora kinases universally regulate the progression of prophase I to diplotene and to the first metaphase. This process, known as the G2/MI transition, involves stepwise regulation of the disassembly of SC, redistribution of SCP3, chromatin remodeling, and condensation of morphologically distinct bivalents [16]. This unique process, which was discovered to be essential to proper recombination and meiotic progression, is a specific ubiquitination of axis proteins and their further regulation by ubiquitin–proteasome system (UPS) degradation. Precisely timed ablation secures proper crossover and prophase I progression [17].

Poly-ubiquitination is a process where Ubiquitin protein chains conjugate to the lysine residues of proteins designated for protein degradation and is implicated in various aspects of meiotic prophase I [17,18,19]. Poly-ubiquitinated substrate proteins are degraded by the 26S proteasome [20]. The ubiquitination process requires three types of enzymes: the ubiquitin-activating enzyme (E1), ubiquitin-conjugating enzyme (E2), and the ubiquitin ligase enzyme (E3). UPS involvement in meiotic events recently described by [21] in yeasts and by [17] in mammalian spermatogenesis point to an extremely important role of the UPS in the epistatic regulation of meiotic proteins for successful prophase I progression. For the first time, these two independent groups showed the localization pattern of the UPS in meiotic nuclei. They observed localization of the majority of ubiquitin foci to the axes of the synaptonemal complex (SC) between homologs. Abundant recruitment of proteasomes along axes also occurred during zygonema and persisted throughout pachynema and diplonema, when chromosomes de-synapsed [17,21,22,23,24]. However, the mechanism remains elusive. 

Vinculin (VCL) is an actin-binding protein, which localizes in the focal adhesion of somatic cells [25,26]. Actin, its interacting partners, and actin-related proteins play important roles in cell nuclei [27,28,29,30,31,32]. VCL has no enzymatic activity, but it regulates cell adhesion by directly binding to actin, stimulating actin polymerization and recruiting actin remodeling proteins [33,34]. While VLC binds to a number of cytoskeletal proteins, it also associates with phosphatidylinositol 4,5-bisphosphate (PIP_2_), which enables its membrane association and thus activation [35]. PIP_2_ is a known factor which induces F-actin polymerization at the inner leaf on a cytoplasmic membrane. Our group has recently discovered that PIP_2_ and actin regulators are also important factors in determining nuclear architecture [36,37]. VCL plays a critical role in mechano-transduction by stabilizing adhesions in response to force. VLC also regulates the dynamics of adhesion at the leading edge of migrating mesenchymal cells and thus mediates the transmission of traction forces [33,38,39,40]. VLC has a critical role in mammalian physiology, its depletion or dysfunction dramatically impairing cell–matrix and cell–cell adhesion, which eventually causes progression of metastasis. 

The data presented here show that in prophase I nuclei, VCL localizes along the newly formed SCs and the centromeric region of homologs, from zygotene to diakinesis. To specify the role of VCL during mouse spermatogenesis, a transgenic mouse strain was prepared with targeted conditional depletion of VCL only in the primary spermatocytes. This enabled us to study the effect on meiotic progression, without affecting the somatic cells [41,42]. It was found that VCL-depleted primary spermatocytes were defective in SC disassembly, causing arrest at the end of prophase I progression. Co-immunoprecipitation of VCL with associated proteins from the nuclei of primary spermatocytes revealed components of the UPS and SUMOylation process, suggesting that VCL might be involved in UPS machinery during prophase I. The present study delivers information about the mechanistic function of nuclear VLC in chromosomal pairing and synapsis and thus enables new perspectives for future studies.

## 2. Materials and Methods

### 2.1. Animals

All males used in the experiments were young adults, i.e., 6–10 weeks old, unless otherwise specified in the text. All mice were bred and raised at the animal facility of BIOCEV and the animal facility of the Institute of Molecular Genetics, AS CR. The protocols for their care and use were approved by the institutional review committee. 

### 2.2. Generation of Vinculin Conditional Knock-out Mouse (VCL cKO) and Genotyping

To produce offspring homozygous for the deleted exon 3 (designated VCL cKO), homozygous Vcl^fl/fl^ (129/Sv mouse) [41] mice were mated to mice which harbored the conditional Smc1β^iCre^ [43,44]. Conditional knock-out males were of genotype Smc1β^iCre^Vcl^fl/fl^. If it is not specified elsewhere, control males were Vcl^fl/fl^ (designated WT). The progeny was genotyped using the following primers. To assess floxed allele, primers Flox Fw (5′ TCAGACCCATACTCGGTTCC 3′) and Flox Rev (5′ AAACTCACAGAGACCCTCCT 3′) were used to produce a 580 bp product. To assess for Cre recombinase expression, primers Cre Fw (5′ AAGCTACAGCGCCGAGAAGCA 3′) and Cre Rev (5′ GAGATGTCCTTCACTCTGATTC 3′) were used to produce an 833 bp Cre product. Cre-mediated excision of VCL exon 3 was confirmed by PCR using primers rec Fw (5′ TTACGCCTAGCACTTGAA 3′) and rec Rev (5′ TGCTCACCTGGCCCAAGATTCTTT 3′) with the following products: recombinant allele—750 bp product, wild-type allele—1400 bp product, and floxed allele—1641 bp product.

### 2.3. Testes Cryo-Sections and Spermatocytes Cell Spreads 

Adult male mice (6–10 weeks old) were euthanized by cervical dislocation. For cryosections, dissected testes were immediately frozen in TissueTek—O.C.T. Compound (SaKuRa Finetek, Torrance, CA, USA) and stored at −80 °C for not more than 1 month. Cryo-sections were 5 μm-thick and transferred on the Fisherbrand™ Superfrost™ Plus Microscope Slides (Thermo Fisher Scientific, Waltham, MA, USA) and fixed immediately in 4% of paraformaldehyde (37%, histology grade, Sigma Aldrich, St. Louis, MO, USA) water solution for 20 min. To spread spermatocytes, dissected testes were decapsulated and processed as previously described [45], with minor modifications. Briefly, seminiferous tubules from a decapsulated testicle were drained in 1 mL of PBS and mechanically disintegrated by 1 min of shaking. The tubules were allowed to sit by gravitation, then we discarded the supernatant and repeated the process. In the following step, PBS was added and incubated for 10 min at room temperature to release SCs. Glass slides were dripped with 20 μL drops of 1% paraformaldehyde (pH 9.2) in PBS with 0.5% Triton X-100 and a cocktail of protease inhibitors (Roche, Hoffmann-La Roche, Basel, Switzerland). The supernatants from the tubules (containing spermatocytes) were transferred into new tubes, cells were pelleted (10 min at 300 g) and resuspended in 200 μL of fresh PBS with proteinase inhibitors and 40 μL of 0.1 M sucrose. Pelleted cells were resuspended by tapping, and 20 μL aliquots of cells were placed onto prepared paraformaldehyde drops on the glass slide. Samples were incubated in a humid chamber for 2 h to let the cells spread on the surface of the glass slide. The samples were then gently washed with PBS with 0.1% Tween and used for immunostaining. 

### 2.4. Seminiferous Tubules Squash

Testes from 6–12 weeks old males were dissected and decapsulated. Testicular tissue was mechanically disturbed by vigorous shaking in 5 mL of PBS or EKRB for at least 1 min. S. tubules were then transferred into a Petri dish. The method for s. tubules squashing and immunofluorescence was described previously [46]. Briefly, isolated s. tubules were transferred to freshly prepared 2% formaldehyde in PBS containing 0.1% Triton X 100 and fixed for 20 min. Separate tubules were transferred by tweezers onto a microscopy slide coated with 1 mg/mL poly-L-lysine (Sigma Aldrich, St. Louis, MO, USA), covered with a coverslip, and squashed by pressing the coverslip. The coverslip was then gently removed, and the samples on the slides were washed four times for 10 min in PBS and immediately used for immunofluorescence.

### 2.5. Immunofluorescence and Microscopy 

Cells on the glass slide were permeabilized in 0.5% Triton-X in PBS for 20 min. After washing the samples in PBS, we blocked the samples in 1% solution of Bovine serum albumin in PBS with 0.1% Tween for 1 h at room temperature or overnight at 4 °C. In this study, the following primary antibodies were used for immunodetection of desired proteins with indicated dilutions (in AbDil buffer = 1% BSA in PBS with 0.1% Tween: rabbit anti SYCP1 (1:300, ab15087, Abcam Cambridge, UK), mouse anti SYCP3 (1:50, sc 74569, Santa Cruz, Dallas, TX, USA), CREST human antiserum (1:100, 15 235 0001 Antibodies Incorporated, Davis, CA, USA), goat anti VCL (1:100, sc 74569, Santa Cruz), rabbit anti SYCP1 (1:400, ab91459 Abcam), mouse anti VCL (1:250, sc 73614 AF647, Santa Cruz), rabbit anti CENP A (1:100, C51A7, Cell Signaling Technology, Danvers, MA, USA), rabbit anti REC8 (1:400, ab149221 Abcam), mouse anti αTubulin (1:100, ab7750 Abcam), rabbit anti-PSMB1 (1:100, PA5–49648 Thermo Fisher), and rabbit anti-Sumo2 + Sumo3 (1:200, ab3742 Abcam). The slides were then washed four times in PBS and incubated with secondary antibodies against mouse, rabbit, goat, and human IgG, and conjugated to Alexa Fluor 488, 568, and 647 (1:400, Thermo Fisher Scineitific). Secondary antibodies were applied to the samples for 2 h when incubated at room temperature or overnight at 4 °C. Detection of polymeric actin was performed by Alexa Fluor^TM^ 568 Phalloidin (1:500, A12380 Thermo Fisher Scientific). Samples were mounted in VectaShield + DAPI anti-fade medium (Vector Laboratories, Newark, CA, USA) or ProLong Gold anti-fade reagent (Molecular Probes). Nuclear spread images were acquired with a Zeiss Axio Imager 2 (Zeiss) or DeltaVision OMX 3D SIM super resolution (GE Healthcare, Chicago, IL, USA). Images of squashed spermatocytes were captured by the STED module on a Confocal LeicaSP8 (Leica, Wetzlar, Germany). Projection of the images, quantification of signal intensity, and measurement of kinetochore distances were performed in the image-processing program ImageJ (Java) or the ZEN 2 blue edition image software (Zeiss, Oberkochen, Germany).

### 2.6. TUNEL Assay

The TUNEL assay identifies apoptotic germ cells. Testes cryo-sections were used to detect apoptotic cells (see protocol above). After fixing the tissue sections, the apoptotic cells were stained with digoxigenin-labeled poly(A) nucleotide probes from an ApopTag Plus Fluorescein In Situ Apoptosis Detection Kit according to the manufacturer’s recommendations (S7111, Merck Millipore, Burlington, MA, USA).

### 2.7. Short-Term Culture of Spermatocytes and Okadaic Acid Treatment

Short-term culture of spermatocytes was performed as previously described [47]. Okadaic acid (OA, Biotech/Cell signaling) was added to a final concentration of 5 µM, while equivalent volumes of 1 M DMSO were added to “no treatment” control cultures. Both short-term cultures of spermatocytes were incubated for 6 h at 32 °C in 5% CO_2_. Cells were pelleted at 1600 rpm for 5 min, washed two times in PBS, and processed for spermatocyte spreading and immunostaining as described above [45]. 

### 2.8. Testicular Single-Cell Suspension, Sperm Count, and Sperm Head Evaluation

Testicular single-cell suspension was produced according to the protocol for sorting according to Bastos et al. [48], with minor modifications. Briefly, testes from euthanized males were decapsulated, and seminiferous tubules were drained in EKRB (enriched Krebs-Ringer bicarbonate medium) and disintegrated by vigorous shaking for at least 1 min. Collagenase IV was added at a final concentration of 0.5 mg/mL (origin Clostridium Histolyticum, Sigma Aldrich) and incubated for 20 min at 32 °C in a thermo block. The tubules were disintegrated by slow pipetting of the sample with a serological pipet. One ml of EKRB with collagenase was added, and incubation was repeated. The sample was filtered through a 100 μm cell strainer (BD Falcon) and washed twice in EKRB with 1% FCS. After final cell sedimentation at 300 g for 8 min, the cells were diluted in 500 μL of EKRB with 1% FCS. The sperm count was evaluated on sperm taken from dissected epididymis. Briefly, cauda epididymis was cut and immersed in 200 μL of EKRB buffer and incubated from 30 min at room temperature. Twenty microliters of supernatant was transferred to the Bürker Counting Chamber, and sperm counts were acquired by Zeiss AxioZoom.V16 macroscope. Next, sperm were dropped on the glass slide, fixed with 4% formaldehyde, and stained with DAPI. From microscopy pictures, we measured at least 100 WT and 100 VCL^cKO^ sperm heads to determine their length and width, according to standard methodology [49]. 

### 2.9. FACS of Tubular Cells and RT-qPCR

Primary and secondary spermatocytes, round spermatid, and sperm, in addition to Sertoli cells and spermatogonia stem cells, were FACS-sorted from single-cell suspension, stained with Hoechst 33342 (5 μg/mL) for 1 h at 32 °C, and then immediately transferred to ice. Propidium iodide was added before FACS analysis at a final concentration of 2 μg/mL to mark dead cells. Individual populations were identified according to Hoechst red and blue emission [48]. Sorted cells were dropped immediately into an RLT buffer with B-mercapto-ethanol and processed for RNA isolation according to the manufacturer’s recommendations (RNeasy Micro Kit, QIA Gen). Primers used in this study were as follows: VCL fw: 5′ ATGCCAGTGTTTCATAC 3′; VCL rev: 5′TCTAGATCCGGTGGATCC 3′; CASC3 fw: 5′ TTCGAGGTGTGCCTAACCA 3′, CASC3 Rev: 5′ GCTTAGCTCGACCACTCTGG 3.

### 2.10. Colchicine Treatment and Chromosome Segregation Analysis

MI and MII preparations were made from four 12-week-old males of wild type and VCL cKO genotype. The mice were injected intraperitoneally with 0.1 mL of 0.5% colchicine (Sigma) and euthanized after 2 h of treatment. Testes were dissected and decapsulated, and a single-cell suspension was prepared by incubation of seminiferous tubules in EKRB with Collagenase IV (50 μg/mL of suspension, Signa Aldrich) for 20 min. The cell suspension was filtered through the cell strainer (70 µm), and cells were pelleted by centrifugation (10 min at 300 *g*). Cells were resuspended in freshly prepared hypotonic solution of 0.56% KCl, fixed with methanol glacial–acetic acid (ratio 3:1), and dropped onto a poly-L-lysine treated glass slide. The dry sample was gently washed with PBS and used for immunofluorescence. The frequency of the univalent was scored for 30 M*I* cells per male, and the presence of monad chromosomes as specified for 20 M*II* cells per male [50,51].

### 2.11. Western Blot Protein Analysis

Proteins were extracted from single germ cells in the lysis buffer (150 mM NaCl, 5 mM EDTA pH 8.0, 50 mM Tris.Cl pH 8.0, 1% NP-40, 0.5% Na-deoxycholate, 0.1% SDS) Twenty microliters of 1 mg/mL protein extract was loaded per lane on SDS polyacrylamide gels. For protein separation, homemade 10% SDS-PAGE gels were used. After separation, proteins were transferred to a PVDF Western blotting membrane (Roche) with a wet Western blotting apparatus (Labnet). Blots were probed with primary antibodies overnight at room temperature in PBS with 1% BSA. The following primary antibodies were used: rabbit anti VCL antibody (ab91459, Abcam) and mouse anti αTubulin (ab7291, Abcam, 1:1000). After 4 × 5 min of washing in PBS with 0.1% Tween 20, membranes were incubated with desired secondary Irdye-conjugated antibody diluted in PBS with 0.1% Tween 20 buffer, goat anti-rabbit Irdye 680RD (ab216779 Abcam, 1:10,000) and goat anti-mouse Irdye 800RD (ab216774 Abcam, 1:10,000), for 1 h on roller at room temperature or overnight at 4 °C. After washing in PBS, the blots were scanned with an Odyssey infrared imaging system (LI COR).

### 2.12. Preparation of Cell and Nuclear Protein Extracts 

To identify VCL-interacting partners, the whole-cell (Cl) and nuclear (Nu) extracts were prepared from testicular single-cell suspension (T-Cl, T-Nu) or FACS-sorted primary spermatocytes (SC-Cl, SC-Nu). For total cell lysate, the RIPA buffer was added into the pelleted testicular cells from single-cell suspension (T-Cl), or direct FACS sorting was carried out to achieve the desired population of primary spermatocytes in an ice-cold RIPA buffer (SC-Cl). Lysate was sonicated on ice for 1 min at a 180-watt power (in rounds of 10 s sonication/10 s rest for each cycle). The protocol for nuclei isolation was adopted from Matunis et al., with minor modifications [52]. Briefly, cells (from single-cell suspension or FACS-sorted) were mechanically disturbed by grinding in a 2 mL glass vessel homogenizer chilled on ice, with buffer A (0.25 M sucrose, 50 mM Tris–HCl (pH 7.5), 25 mM KCl, 5 mM MgCl_2_, 2 mM DTT, 1× protease inhibitors). Suspension was filtered through a 25 μm cell strainer, and obtained nuclei were sedimented by centrifugation at 800 *g* for 20 min at 4 °C. During the centrifugation, 5 mL of buffer B (2.3 M sucrose, 50 mM Tris–HCl (pH 7.5), 25 mM KCl, 5 mM MgCl_2_, 2 mM DTT, 1× protease inhibitors) was added to the bottom of 6 Beckman SW28 ultracentrifuge tubes. Pelleted, rough nuclei were re-suspended in 500 uL of ice-cold B buffer and gently transferred to the B-buffer surface in the tube. Samples were centrifuged at 27,000 rpm (141,000 g) in a Beckman SW28 rotor for 1 h at 4 °C. Clean nuclei either from the whole testes (T-Nu) or primary spermatocytes (SC-Nu) appeared as translucent pellets at the bottom of the tube. The nuclei were immediately resuspended in a lysis buffer and sonicated as described above. Lysates were used directly for co-immunoprecipitation.

### 2.13. Co-Immunoprecipitation and Protein Digestion

Co-immunoprecipitation (Co-IP) was performed by a specific, commercially available polyclonal anti-Vinculin antibody (ab91459, Abcam). After overnight incubation with antibody at 4 °C, 5 (T-Nu, SC-Nu) or 20 μL (T-Cy, SC-Cy) of Dynabeads with conjugated Protein A (Dynabeads Protein A Immunoprecipitation Kit, Novex) was added, and the procedure was carried out according to the manufacturer’s recommendations. In the control experiment, the rabbit IgG polyclonal isotype control (ab37415, Abcam) was applied to determine the non-specific proteins bound to the beads. Co-IP samples were resuspended in 100 mM TEAB containing 2% SDC. Cysteines were reduced with a 10 mM final concentration of TCEP and blocked with a 40 mM final concentration of chloroacetamide (60 °C for 30 min). Samples were cleaved on beads with 1 µg of trypsin at 37 °C overnight. After digestion, the samples were centrifuged, and the supernatants were collected and acidified with TFA to a final concentration of 1%. SDC was removed by extraction to ethyl acetate [53]. Peptides were desalted using in-house-produced stage tips packed with C18 disks (Empore) according to Rappsilber et al. [54]. 

### 2.14. nLC-MS 2 Analysis and Data Analysis

A nano reversed phase column (EASY-Spray column, 50 cm × 75 µm ID, PepMap C18, 2 µm particles, 100 Å pore size) was used for LC/MS analysis. Mobile phase buffer A was composed of water and 0.1% formic acid. Mobile phase B was composed of acetonitrile and 0.1% formic acid. Samples were loaded onto the trap column (Acclaim PepMap300, C18, 5 µm, 300 Å Wide Pore, 300 µm × 5 mm, 5 Cartridges) for 4 min at 15 μL/min. The loading buffer was composed of water, 2% acetonitrile, and 0.1% trifluoroacetic acid. Peptides were eluted with Mobile phase B gradient from 4% to 35% B in 60 min. Eluting peptide cations were converted into gas-phase ions by electrospray ionization and analyzed on a Thermo Orbitrap Fusion (Q-OT- qIT, Thermo). Survey scans of peptide precursors from 350 to 1400 *m/z* were performed at 120 K resolution (at 200 *m*/*z*) with a 5 × 105 ion count target. Tandem MS was performed by isolation at 1.5 Th with a quadrupole, HCD fragmentation with a normalized collision energy of 30, and rapid scan MS analysis in the ion trap. The MS/MS ion count target was set to 104, and the max injection time was 35 ms. Only those precursors with a charge state of 2–6 were sampled for MS/MS. The dynamic exclusion duration was set to 45 s with a 10 ppm tolerance around the selected precursor and its isotopes. Monoisotopic precursor selection was switched on. The instrument was run in top speed mode with 2 s cycles [23]. All data were analyzed and quantified using the MaxQuant software (version 1.5.3.8) (Max-Planck-Institute of Biochemistry, Martinsried, Germany) [55]. The false discovery rate (FDR) was set to 1% for both the proteins and peptides, with a specified minimum peptide length of seven amino acids. The Andromeda search engine was used for the MS/MS spectra search against the Mus musculus database (downloaded from uniprot.org in February 2015, containing 44,654 entries). Enzyme specificity was set as C-terminal to Arg and Lys, allowing for cleavage at proline bonds and a maximum of two missed cleavages. Dithiomethylation of cysteine was selected as a fixed modification. N-terminal protein acetylation and methionine oxidation were selected as variable modifications. The “match between runs” feature of MaxQuant was used to transfer identifications to other LC-MS/MS runs based on their masses and retention times (maximum deviation 0.7 min), and this was also used in quantification experiments. Quantifications were performed with the above-mentioned label-free algorithms. Data analysis was performed using Perseus 1.5.2.4 software (Max-Planck-Institute of Biochemistry, Martinsried, Germany) [56].

### 2.15. Computational and Statistical Analysis

Microscopy images were processed and quantified using FiJi ImageJ software and appropriate plugins. Charts and statistical analysis were carried out in GraphPad Prism software. Raw data from flow cytometry and FACS analysis and sorting were processed in a FlowJo software package.

## 3. Results

### 3.1. VCL localizes to Meiotic Nuclei during Prophase I

**Figure 1:**
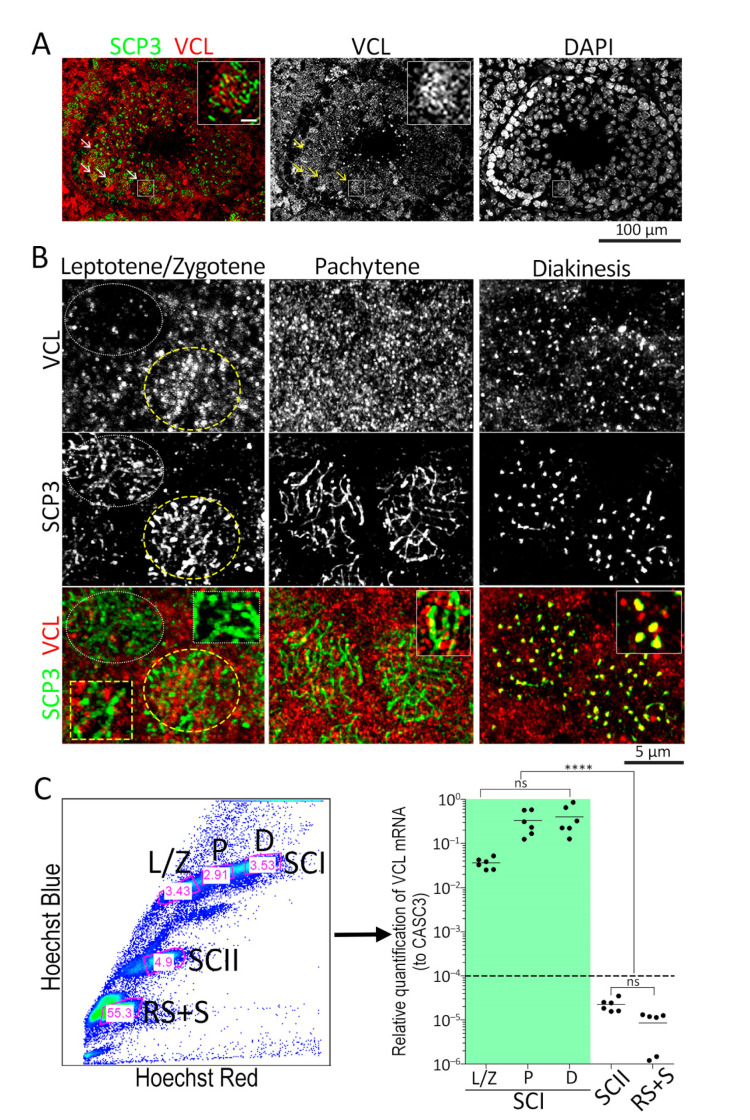
VCL in the nuclei of meiotic cells. (**A**) Immunofluorescence staining on 5 µm-thick sections of testes sections revealed VCL localization not only in Sertoli cells (tubular signal) but also at the area of primary spermatocytes (arrows; figure inset, scale bar = 5 µm); (**B**) detailed observations of VCL localization of testes sections, where leptotene SCs (dotted line) contain no VCL, which appears first in zygotene (yellow dashed line) remaining until diakinesis; (**C**) single-cell suspension indicated with Hoechst 33342 and measured emissions in red and blue light spectra, resulting in a typical scythe-like profile of spermatocyte populations. Sorting gates (pink) are depicted in the area of the sorted cells, with indicated perceptual proportion of cell content for all cells. Spermatogenic cells were FACS-sorted according to their ploidy and used for VCL mRNA relative quantification (comparing the expression level to house-keeping CASC3). VCL expression peaked in the pachytene and diplotene stages (green box). The level of VCL mRNA in other cells was below the limit of positivity (dashed line). Each value represents a biological replicate, i.e., one wild-type male mouse. For statistical evaluation, an unpaired *t*-test was applied, where ns = 0.1744 and highly significant (****) *p* < 0.0001. Key to the figure: SCI = primary spermatocyte; L/Z = leptotene/zygotene; P = pachytene, D = diplotene; SCII = secondary spermatocyte; RS + S = round spermatids and sperm.

In seminiferous tissue, VCL localizes to the apical ectoplasmic specialization [57] and the tubulobulbar complex of Sertoli cells [58]. This is part of the protein complex necessary to support endocytic-vesicle-mediated protein trafficking (together with clathrin, N-WASP, cortactin, zyxin, Arp3, and Eps8 [58,59,60,61]. The VCL was found not only in the Sertoli cytoplasm but also in the area where early spermatocytes localize (Figure 1A), colocalizing VCL with SCP3—a synaptonemal complex protein. In detailed observation, VCL was found in the nuclei of prophase I spermatocytes (Figure 1A inset and Figure 1B). A weak or no VCL signal was present in leptotene spermatocytes and was next detectable in zygotene SCs, but no colocalizing with SC occurred, which is indicated herein by SCP3 (PCC = 0.0023). VLC first formed foci in pachytene spermatocytes and decorates fully formed SC (Figure 1B, pachytene, PCC = 0.1546). In diakinesis, VLC formed distinct clusters, which co-localized with the centromeric region with preserved SCP3 (Figure 1B, PCC = 0.7534). In the next step, we quantified VCL mRNA from FACS-sorted spermatocytes (Figure 1C). The Hoechst-33342 flow cytometric method was used to isolate individual spermatocyte populations from the adult murine testis on the testicular single-cell suspension. Hoechst emission in the blue and red spectra of light allows identification of spermatogenic cells in meiosis I, meiosis II, or final-round spermatids and sperm [62]. The desired cell populations were gated (Figure 1C, left panel), and the cells were sorted directly into the lysis buffer for further isolation of mRNA. The qPCR reaction with specific VCL primers showed more VCL mRNA in primary spermatocytes (SCIs) than secondary spermatocytes (SCIIs) or round spermatid and sperm (RS + S) (Figure 1C, right panel). From each cell population, we sorted at least 10,000 cells for isolation and quantification of mRNA. Early VCL mRNA was detectable in the mixed population of leptotene and zygotene spermatocytes (L/Zs), but these were indistinguishable from each other in this experiment. More interesting was the high expression of VCL in pachytene and peeking in diplotene SCs, which were relatively quantified to the house-keeping gene (Figure 1C, green box in the right panel).

**Figure 2:**
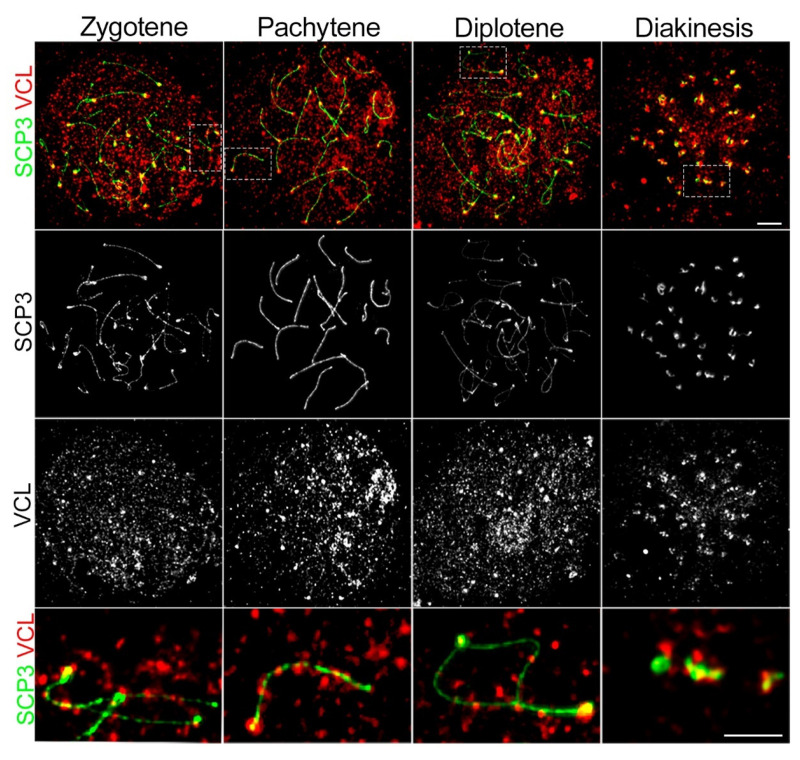
Subnuclear localization of VCL during prophase I in the spermatocyte chromosomal spreads. Immunofluorescence of spermatocyte spreads from adult mouse males shows localization of VCL (red) and SCP3 (green). Data indicate dynamic changes in VCL localization during meiotic prophase I substages. VLC started to accumulate in the zygotene stage. In pachytene, VCL localized in defined foci near the centromeric region and decorated paired homologous chromosomes. The decorating pattern dissolved during the diplotene substage, whereas obvious centromeric localization remained until the diakinesis substage. Scale bars correspond to 10 µm.

To better visualize the VCL pattern in the primary spermatocytes of the testes sections, VCL was localized on spermatocyte spread samples [63]. Prophase I staging was performed according to SCP3 localization patterns. In zygotene, VCL formed foci in the close vicinity of the centromere termini of sister bivalents (Figure 2, zygotene), whereas in pachytene, VCL redistributed and obviously decorated fully associated chromosome axes where the synaptonemal complex was organized (Figure 2, pachytene). This decoration remained in diplotene in the area of homologous tetrads (Figure 1, diplotene, inset) and interestingly remained in the area of the centromere. In diakinesis, VCL accumulated on the termini of the chromosome tetrad (Figure 2, diakinesis), colocalizing with SCP3, which was already found on tissue sections (Figure 1B, diakinesis). 

**Figure 3:**
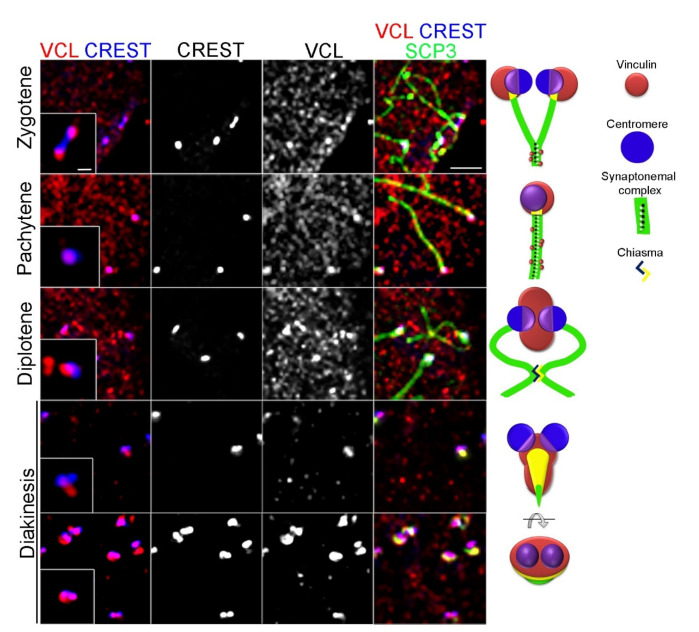
Co-localization of VCL and CREST-labeling centromeres on squashed spermatocytes. Immunofluorescence. VCL (red), CREST (blue), and SCP3 (green) were immunodetected in the primary spermatocytes. The meiotic stage was specified according to the state of chromosome pairing, i.e., pattern of SCP3. We found diverse VCL morphology regarding prophase I progression, associated with centromeres. We analyzed more than 100 squashed spermatocytes of each prophase I stage. The panel to the right depicts a localization pattern and represents the most observed conditions in each stage. The scale bar corresponds to 10 µm. The scale bar in the inset corresponds to 2 µm.

An interesting pattern of VCL localization to the chromosome termini was further specified by co-localizing VCL with centromeres (CREST). A specific and statistically reproducible pattern of VCL appearance, with prophase I progression, was found in this region. In zygotene, VCL associated with the centromeres of as yet un-paired centromeres (Figure 3, zygotene). In pachytene, it entirely colocalized with the centromere of fully synapsed chromosomes (Figure 3, pachytene). In the diplotene stage, VCL changed shape and was present in a “bean like” structure, which kept the centromeres attached, whereas bivalent splitting was limited only by cross-over and associated centromeres. In the following diakinetic stage, a “bean-like” VCL pattern remained, with attached centromeres (Figure 3, diakinesis). We found the condition where the centromeric aggregate was captured from the top, showing the VCL disk co-localizing with a centromeric CREST (Figure 3, diakinesis, bottom panel). The observed VCL pattern seems to be dynamic, and the most common VCL pattern in each meiotic stage is depicted (Figure 3, right panel). 

### 3.2. Depletion of VCL in Primary Spermatocytes Causes Decreased Fertility in Males 

**Figure 4:**
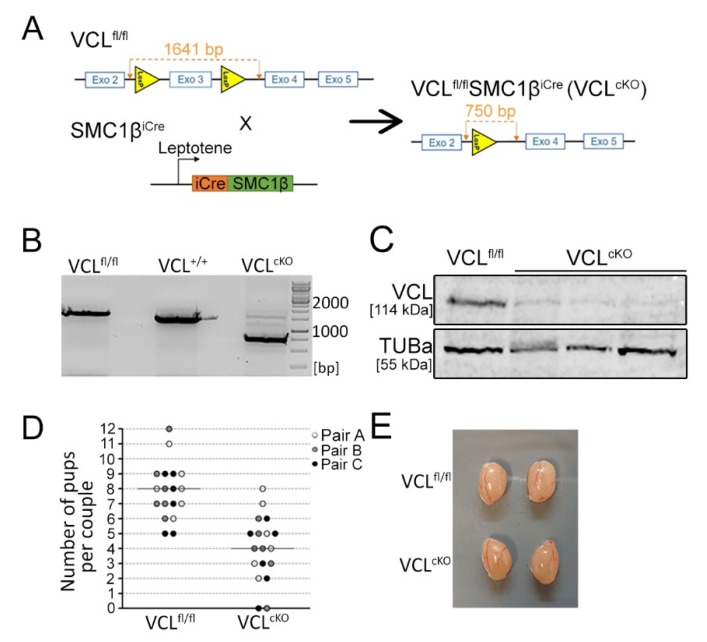
Confirmation of VCL depletion. Cre-mediated excision of VCL exon 3 was confirmed by PCR with the following products. (**A**) Mice with two genotypes were cross-bred, gaining VCL conditional knock-out (VCL^cKO^) in primary spermatocytes. Using the scheme, the size of the PCR product of the floxed 3rd VCL exon (i.e., VCL^fl/fl^) was determined, which is 1641 bp. After Cre-mediated excision, the final PCR product 750 bp was obtained, confirming the depletion of exon 3; (**B**) PCR genotyping of VCL-flox VCL allele (VCL^fl/fl,^, 1641 bp), wild-type VCL (VCL^+/+^, 1500 bp), and knocked-out allele (VCL^cKO^, 750 bp); (**C**) depletion of VCL on the protein level was confirmed by the Western blot from the testicular protein extract. A testicular lysate from three VCL^cKO^ individuals was observed; (**D**) breeding performance of VCL^cKO^ males. The progeny was counted from four breeding pairs of both male genotypes—VCL^fl/fl^ and VCL^cKO^. Each male was bred with two WT females for at least 6 months. The decreased number of pups is evident. In the case of the VCL^cKO^ male from the three evaluated parent pairs, the average number of pups was 4, compared to 8 in the WT pair; (**E**) the size of testes between the VCL^fl/fl^ and VCL^cKO^ males was compared, and no significant difference was found.

To test the direct effect of VCL on meiotic progression, a transgenic mouse model was prepared, with VCL conditional knock-out (VCL^cKO^). To acquire this, we took advantage of Aire^fl/fl^ mice previously generated by Zemljic-Harpf et al. [41]. As described previously, loxP sites contain VCL’s exon 3, which has been shown to be sufficient in inactivating VCL from targeted cells [41]. The VCL^fl/fl^ females were cross-bred with transgenic males carrying iCre recombinase expressed under the SMC1β promoter (SMC1β^iCre^), active from leptotene in prophase I (Figure 4A). The SMC1β protein is a meiosis-specific component of the cohesion complex, localizing on the chromosomal axes from the leptotene and remaining until the metaphase II–anaphase II transition [47,48,64]. In the resulting VCL^fl/fl^SMC1β^iCre^ males, VCL^cKO^ and also sequencing (data not shown) confirmed the deletion of VCL exon 3 by PCR (Figure 4B). Depletion of the VCL protein was also shown in the protein lysate obtained from primary spermatocytes. SDS-PAGE, followed by detection of VCL on Western blot with a specific anti-VCL antibody, did not detect any protein in the cell lysate prepared from three individual VCL^cKO^ males (Figure 4C). Direct evidence that VCL might be involved in spermatogenesis and thus supports fertility was tested in a breeding performance experiment (Figure 4D). VCL^cKO^ males were bred with wild-type female for at least 6 months. We regularly checked the number of pups and compared it to wild-type couples breeding at the same time. Significantly fewer pups from VCL^cKO^ male breeding were observed (Figure 4D). This trend was observed throughout the breeding period. Next, the testes’ size was checked, but there we found no significant deviation compared to VCL^cKO^ and wild-type controls of the same age (Figure 4E). In addition, epididymal sperm counts were performed on epididymal sperm of WT and VCL^cKO^ males. A significant decrease in the number of sperm was observed in VCL-depleted males (Supplementary Figure S1A). However, VCL^cKO^ males still contained a sufficient number of sperm for fertilization. Sperm head morphology was not affected in VCL^cKO^ males. Next, the sperm head morphology and formation of acrosome were analyzed. The length and width of the sperm head were measured. There was no significant deviation from either parameter in VCLcKO sperm, compared to the control sample (Supplementary Figure S1B). Structural or functional acrosomal abnormalities could impair sperm fusion, and ultimately result in infertility (reviewed in [65]). VCL^cKO^ males possess a wild-type-like acrosome properly formed on the dorsal area of the sperm head (Supplementary Figure S1C). Regarding this observation, it is more likely that in VCL^cKO^ males, the process of spermatogenesis is affected rather than spermiation.

### 3.3. Depletion of VCL Causes Unwanted Centromeric De-Synapsis in Diplotene

**Figure 5:**
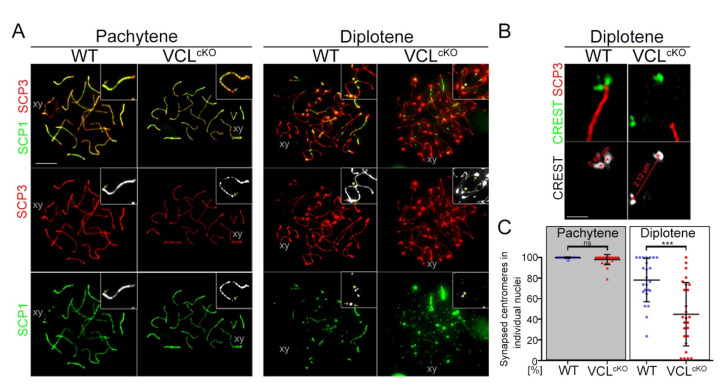
VCL^cKO^ possessed defective synapsis I pachynema and diplonema; (**A**) the patterns of the SCP3 (the axial element) and SCP1 (the central element) of the synaptonemal complex differed between WT and VCL^cKO^. In VCL^cKO^ pachytene nuclei, an irregular SCP1 signal was apparent along the chromosome axis and mostly missing at the centromere (pachytene VCL^cKO^, arrow). In diplotene, SCP1 was usually present only at the associated centromeres (diplotene WT, arrows) and the chiasma (diplotene WT, asterisk). In VCLcKO spermatocytes, centromeres lacked SCP1 and were de-synapsed (diplotene VCL^cKO^, arrows). Strong SCP1 aggregate was found only at the chiasma (diplotene VCL^cKO^, asterisk). The scale bar represents 5 µm; (**B**) staining the centromeres with CREST (green) and SCP1 (red) enabled evaluation of de-synapsis in diplotene VCL^cKO^ spermatocytes. Evaluation of the synapsed and de-synapsed centromeres was performed according to the distance between two CREST foci, i.e., ≤0.6 µm is synapsed and >0.6 µm is de-synapsed. The scale bar represents 1 µm; (**C**) the graph shows the levels of centromere synapsis in each individual nucleus (20–25 autosomes were analyzed) at the pachytene and diplotene in WT and VCL^cKO^. *p*-values represent a comparison of the synapsed groups (Mann–Whitney U-test) for pachytene (ns = 0.0554) and diplotene (*** = 0.0002). Error bars show the mean ± SD. We scored the number of bivalents with synapsed centromeres and present the results in a graph as a percentage of all bivalents. Each value represents one evaluated spermatocyte spread.

As described above, VCL decorated the synaptonemal complex from zygotene and localized abundantly in the centromeric region during the entire prophase I. Based on these localization data, we studied the effect of VCL depletion on synapsis formation. SC spreads were prepared, and we immunodetected proteins of the synaptonemal complex SCP3, the axial/lateral protein, which localizes between the chromosome homologs [63], and SCP1, which is the central element of the synaptonemal complex stabilizing the homologous tetrad [66]. First, the formation and typical quality of synapsis for each prophase I stage in WT and VCL^cKO^ males were evaluated. Aberrant synapsis between the tetrads from pachytene in VCL-depleted spermatocytes was found. The expected confluent colocalization of SCP3 and SCP1 in the control sample was not apparent in VCL^cKO^ spermatocytes (Figure 5A, VCL^cKO^ pachytene). The SCP1 pattern was interrupted and either weak or entirely missing in the centromeric area (Figure 5A, VCL^cKO^ pachytene, yellow arrow). The typical pattern in the diplotene stage was also altered in VCL^cKO^ spermatocytes. As shown in the control sample, SCP1 was mostly depleted from the synapsis, remaining only in the area of associated centromeres (Figure 5A, WT diplotene, yellow arrow) and chiasma (Figure 5A, WT diplotene, yellow asterisk). By contrast, in VLC-depleted spermatocytes, the SCP1 appeared as intense aggregates, with localization to the chiasmata (Figure 5A, VCL^cKO^ diplotene, yellow asterisk) but not to the centromeric area. Moreover, centromeres without SCP1 were prematurely segregated (Figure 5A, VCL^cKO^ diplotene, yellow arrow). Super-resolution microscopy of spermatocytes spreads was applied to visualize immunodetected synaptonemal component SCP1 and centromeric CREST. The presence of the synapsis and centromere conjunction was studied, comparing WT and VCL^cKO^ spermatocytes in the pachytene and diplotene stages. Scoring of centromeric synapsis and de-synapsis was measured and evaluated according to a previously established centromere displacement standard published by Qiao et al. [12]. The distance between two centromeres, ≤0.6 µm, is considered synapsed, whereas > 0.6 µm is de-synapsed (as shown in Figure 5B). In the spermatocyte spread, the number of bivalents with synapsed centromeres was scored, and all bivalents are expressed in a graph as percentages. Each value represents one evaluated spermatocyte spread. Scores 20–25 were given to the pachytene and diplotene spermatocytes from the WT and VCL^cKO^ males. No significant difference existed between WT and VCL^cKO^ centromeric synapses (Figure 5C); however, a few nuclei contained de-synapsed centromeres. A significant change was apparent in the diplotene stage, which had a highly variable amount of centromeric splitting (median around 44%) per cell (Figure 5C). The distance between the split centromeres was measured, and it was found that the distance between de-synapsed homologous kinetochores significantly increased at the pachytene and diplotene in VCL^cKO^ (Supplementary Figure S2). Based on these data, we concluded that VCL’s presence is essential to maintaining the integrity of centromeric synapsis and preventing premature loss of synapsis.

**Figure 6:**
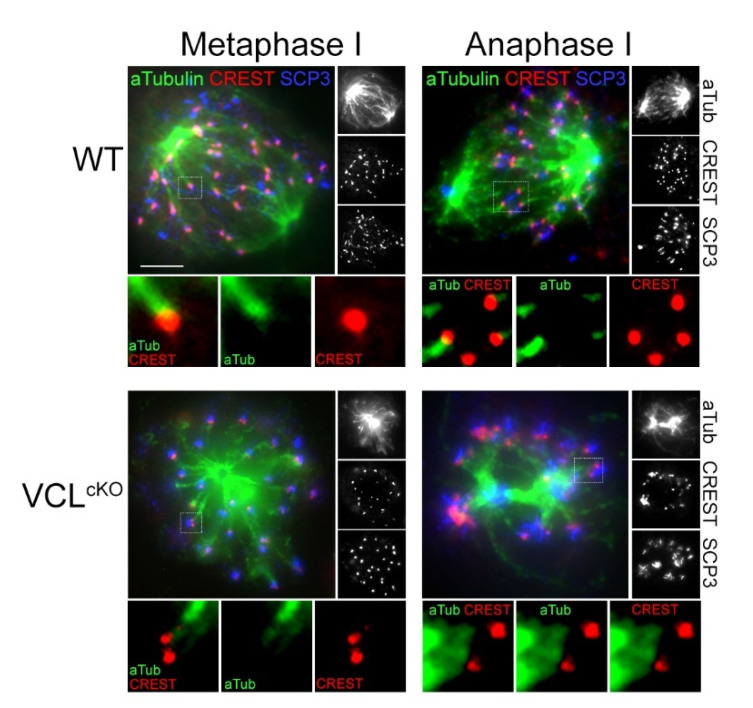
VCL^cKO^ kinetochores were not attached to the meiotic spindle. Metaphase I stage in WT control showed attachment of the kinetochore to the microtubule filament of the meiotic spindle. During anaphase I, sister kinetochores were pulled toward the spindle poles. In the VCL^cKO^ spermatocytes, kinetochores of the bivalent appeared with the spit CREST foci, and microtubule filaments tried to reach each of them (two microtubule filaments, detail on the left). In anaphase I, VCL missing kinetochores was not aligned to the equatorial zone but remain scattered around non-standard meiotic spindle.

During mouse meiosis I, the persistent association of centromeres between homologs and sister chromatids directs attachment to microtubules (MTs) from the opposite poles, the spindle assembly checkpoint (SAC), and triggers chromosomal segregation [13,14,16]. Defective disassembly of the SC at the sites of centromeres and increased distances between kinetochores of aligned bivalents in VCL^cKO^ suggest defects in the formation of the meiotic spindle. To examine this possible scenario, the meiotic spindle of spermatocytes isolated was analyzed using a seminiferous squash assay [46]. Unlike the spermatocyte spreading method, a gentle procedure of seminiferous tubules squash retains cell cytoplasm in an intact state and allows staining of the tubular network [67]. In the WT control, a standard spindle morphology in metaphase I spermatocytes was observed (Figure 6, WT). The kinetochore of two tightly associated bivalents was attached to the microtubule of the spindle (Figure 6, WT, left panel). In anaphase I, the kinetochores were oriented toward the spindle poles, pulled by the tubular force to the sites (Figure 6, WT, right panel). By contrast, VCL-depleted metaphase spermatocytes very often possessed non-standard spindle morphology (Figure 6, VCL^cKO^). Kinetochores were present as two separate CREST foci, which is the consequence of previously shown premature centromere splitting. The microtubules from the spindle tried to reach each part of the kinetochore (apparent as two separate filaments, Figure 6, VCL^cKO^, left panel) but enabled attachment to them. This also affected the anaphase stage, where kinetochores were not attached to the spindle and remained surrounding the collapsed spindle, without desired bipolar orientation (Figure 6, VCL^cKO^, right panel). This interesting finding might relate to a possible new function of VCL in the kinetochore assembly. We note that spindle formation is directly dependent on kinetochore formation. Thus, observation of non-standard spindle formation might be the consequence of missing or abrogated chromosome kinetochores in VCL^cKO^ males.

### 3.4. VCL Is Required for Meiotic Progression

**Figure 7:**
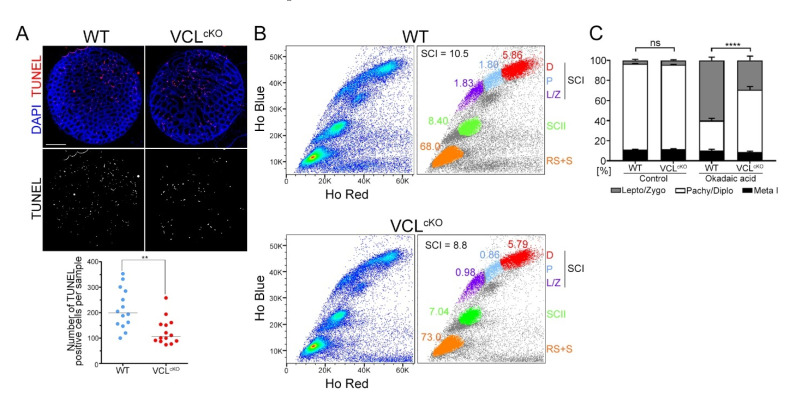
In VCL-depleted testes, spermatocytes underwent meiotic arrest; (**A**) TUNEL assay on the testes sections of WT and VCL^cKO^ testes showing TUNEL-positive cells, i.e., apoptotic cells. We stained 12–15 testes sections with an ApoTag detection kit. The number of red foci (i.e., apoptotic cells) was counted. In the graph below, the number of foci of each testes section is depicted, with an assigned median value. Statistical evaluation involved an unpaired *t*-test, where ** is 0.0016; (**B**) testicular single-cell suspension stained with Hoechst-33342 in two distinct channels classified populations of cells into primary spermatocytes (SCIs), secondary spermatocytes (SCIIs, green), and round spermatids and sperm (n, orange). SCI is composed of three subpopulations: mixed leptotene/zygotene (L/Z, purple), pachytene (P, light blue), and diplotene (D, red). In the right panel, the SCI value indicates the percentage of SCI from all cells. The VCL^cKO^ sample contains fewer SCI. The decrease was apparent in the L/Z (WT = 1.83% to VCL^cKO^ = 0.98%) and P (WT = 1.80% to VCL^cKO^ = 0.86 %) subpopulations, but not in D (WT = 5.86% to VCL^cKO^ = 5.79%). Prophase I arrest affected the count of SCIIs (WT = 8.40% to VCL^cKO^ = 7.04%); (**C**) A short-term culture of VCL^cKO^ and control spermatocytes was treated with okadaic acid (OA) at 5 mM concentration and incubated for six hours. Quantification of G2/MI transition revealed a progression of 60.02% of prophase I spermatocytes to diakinesis/prometaphase I in the treated WT culture. Progression of only 29.41% of pachytene/diplotene spermatocytes was observed in the treated VCL^cKO^ culture. The *p*-value represents a comparison of progression to metaphase I (chi-squared test) between the control and VCL^cKO^. No treatment, ns = 0.7517; OA treatment, **** < 0.0001. Error bars show the mean ± SD.

During meiosis, apoptosis is triggered when defects in complex processes such as synapsis, recombination, and segregation of homologues compromise the quality of the spermatocyte [64]. Indeed, the normal occurrence of these processes is strictly regulated by meiotic checkpoints, which create a network of signaling mechanisms which modulate the activity and progression of meiosis. Depletion of essential survival factors and persistent meiotic defects are detected by these highly conserved meiotic checkpoints, which then cause an arrest of cellular progression of the defective cells and an induction of the apoptotic program [68]. Apoptosis of spermatocytes with timely inappropriate de-synapsis could be the cause of observed decreased fertility in VCL^cKO^ males. Thus, a TUNEL assay was applied to testes sections of WT and VCL^cKO^ males to detect apoptotic cells. To our surprise, significantly fewer positive cells were found in VCL^cKO^ testes sections than in WT controls (Figure 7A). Counting the TUNEL-positive foci revealed that their number in WT varied between the samples, with a median of around 200 foci per section (Figure 7A, microscopy picture and graph). By contrast, the number of foci in VCL^cKO^ testes was low, maintaining a median of 100 foci per sample (Figure 7A, microscopy picture and graph). This observation led us to hypothesize that not dying out but rather meiotic arrest, which is usually a prerequisite for apoptosis, might be the effect of VCL depletion on primary spermatocytes. Analysis of the individual spermatocyte cell population count and quality can be performed using the Hoechst-33342 flow cytometric method [62]. The Hoechst was excited using a 375 nm laser. The dye’s wide emission spectrum was detected in two distinct channels: “Ho Blue” (450/40 nm band-pass filter) and “Ho Red” (670 nm long-pass filter). The redistribution of spermatocytes then appeared according to their ploidy and DNA state, allowing us to distinguish between primary spermatocytes (SCIs), secondary spermatocytes (SCIIs), and round spermatid and sperm (RS + S) (Figure 7B). SCI contains subpopulations of mixed leptotene/zygotene (L/Z), pachytene (P), and diplotene (D) spermatocytes (Figure 7 B). The same number of cells from the WT and VCL^cKO^ was FACS-sorted and showed a clear difference in cell count of SCIs and SCIIs (Figure 7B). In the VCL^cKO^ SCII subpopulation, the reduction in the L/Z (Figure 7B, purple) and P (Figure 7B, light blue) population was evident, but not in D (Figure 7B, red), which is comparable to the WT control. Disproportional SCI cell redistribution is typical for obstructions in prophase I progression, i.e., meiotic arrest. Enrichment in the diplotene sub-population was also confirmed with a frequency visualization plot (Supplementary Figure S3). Arrest of the spermatogenesis at the end of prophase I led to a decreased number of SCIIs (Figure 7B, SCIIs in green). To confirm the hypothesized meiotic arrest, the G2/MI transition of VCL^cKO^ primary spermatocytes was evaluated. Prophase I spermatocytes did not progress to metaphase I ex vivo under standard cell culture conditions. However, G2/MI transition is inducible by okadaic acid (OA) and the PP1 and PP2A protein phosphatase inhibitors [69]. The effect of VCL depletion on G2/MI progression was tested using an OA-treated prophase exit assay (Figure 7C). A short-term culture of VCL^cKO^ and control spermatocytes was treated with OA. Subsequently, microscopy of immunostaining enabled us to distinguish diakinesis and prometaphase I on chromosomal spreads. In this experimental setup, a marker of kinetochores CREST was immunodetected, with SCP3, the lateral element of SC (in diakinesis remaining only at the centromere), and REC8 localizing along the sister chromatid until anaphase I (Supplementary Figure S4) [3,70]. Quantification of G2/MI transition revealed progression of 60% of prophase I spermatocytes to diakinesis/prometaphase I in an OA-treated control culture in contrast to 29% in an OA-treated VCL^cKO^ culture (Figure 7C). This confirms that depleted seminiferous tubules in VCL are primary spermatocytes arrested at diakinesis (at the end of prophase I) and unable to proceed with division. 

### 3.5. VCL Associates with Proteasome Subunits in Pachytene Nuclei

**Figure 8:**
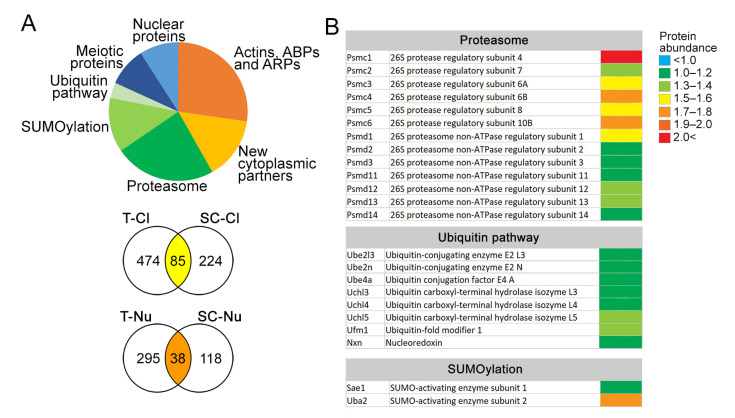
VCL interacted with the components of UPS in meiocytes; (**A**) the graph depicts the group of proteins co-IP-ed with VCL. The number of identified proteins is indicated in the circles, and proteins were found in both sets, i.e., we found 85 common proteins in the cytoplasmic fraction and 38 proteins in the nuclear fractions. These proteins were significantly abundant in the cytoplasmic (Cl) and nuclear extracts (Nu) in both testicular single-cell suspension and FACS-sorted pachytene spermatocytes. The biggest portion of interacting partners were from the cytoskeleton and cytoplasm. However, the majority of the proteins belonged to the UPS; (**B**) the list of co-IP-ed proteins consists of proteasome and its subunits, ubiquitination, and the SUMOylation pathway. Here, only the most abundant proteins are shown. The heat map indicates enrichment (log-fold change) of the representative protein in the co-IP experiment compared to the isotype control experiment.

To understand the molecular mechanism behind decreased fertility and distorted meiotic progress in VCL-deficient males, the VCL spermatogenic interactome was investigated using mass spectrometry. In our experimental setup, we prepared both cytoplasmic (Cl) and nuclear (Nu) extracts from either testicular single-cell suspension (T-Cl, T-Nu) or FACS-sorted pachytene spermatocytes (SCs-Cl, SCs-Nu) (Figure 8A). Here, the significantly enriched co-immunoprecipitated proteins are listed, which were present in both single-cell suspension and also enriched pachynema (Figure 8A). Abundant proteins were divided into groups according to their subcellular localization and pathway (Figure 8B). In addition to the known cytoplasmic-interacting partners (actin, actinin, filamin, paxillin, tubulin, vimentin, and Twinfilin-1), new cytoplasmic partners and proteins associated with meiotic progression were found (Supplementary Figure S5). The most interesting interacting proteins were the components of UPS (Figure 8B). Among these proteins, the ubiquitin conjugation enzymes (Ube2, Ube3), proteins of proteasomal 19S regulatory particles (Psmc1-6, Psmd1-3, and Psmd11-14), and ubiquitin carboxy-terminal hydrolase type 3 and 5 (UCH-L3 and UCH-L5) were the most prominent (Figure 8B). 

**Figure 9:**
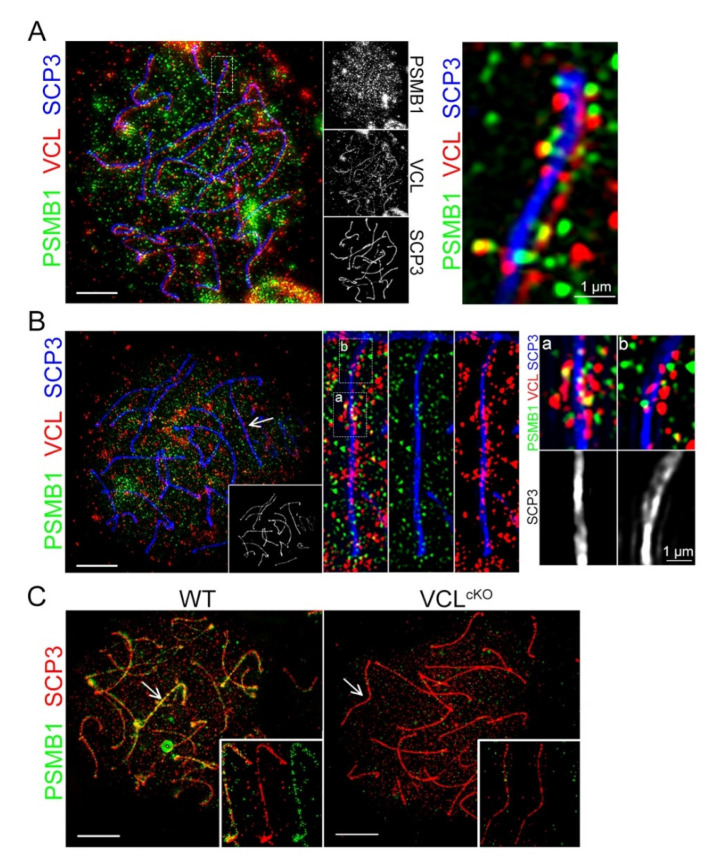
VCL associated with proteasome complexes in the area of the full synapse, and its depletion detached the proteasome from the chromosome axes; (**A**) co-localization of VCL and PSMB in the pachytene spermatocytes was not strong. However, from detailed observation, these two proteins might appear to associate at chromosomal axes; (**B**) VCL/PSMB aggregates appeared more frequently together at the SCP3 full synapsis (a) but not when the SCP3 axes were separate (b); (**C**) the best localization pattern of PSMB in the pachytene spermatocyte visualized by STED. The decorating pattern on the axes was not observed in VCL^cKO^ spermatocytes.

To confirm our MS data, VCL with proteasome was co-localized. For this purpose, an antibody against Proteasome Subunit Beta 1 (PSMB1) was used, which is one of the key subunits of 20S proteasome recently shown to be crucial for proper meiotic progression [17]. Using structured illumination super-resolution microscopy, it was found that VCL clearly associated with PSMB1 exclusively at the synaptonemal complex, indicated herein as SCP3 (Figure 9 A, B, inset a). When chromosome axes were not fully associated, PSMB1 and VCL decorated the synaptonemal complex but did not form a complex (Figure 9B, inset b). This interesting observation led us to hypothesize that VCL might either reposition or stabilize proteasome at the fully formed synaptonemal complex between homologs. Thus, PSMB1 in VCL^cKO^ pachytene spermatocytes was localized. As reported in the publication by Rao et al., proteasomes decorate the pachynema synaptonemal complex before recombination [17]. The pattern on WT pachytene spermatocytes was confirmed, but VCL^cKO^ spermatocytes diminished the majority of the PSMB1 signal (Figure 9C).

Proteasome localization along the chromosome axes was interdependent of SUMO and ubiquitin and mediated largely by E3 ligases (such as RNF212 and HEI10). In the next step, localization and quantification of the SUMOylation signal were determined in the pachytene nuclei with and without VCL (Supplementary Figure S6). The pachytene spermatocyte nucleoplasm was separately analyzed without the area of the sex body, as the non-homologous sex chromosomes in males were shown to behave and be regulated differently (reviewed in [71]) regarding the SUMOylation pathway [17]. The level of SUMOylation signal was significantly reduced in VCL^cKO^ pachytene spermatocytes, especially in the whole nuclear area but also in the nucleoplasm excluded by gonosomal chromatin concentrated in the sex body structure. (Supplementary Figure S6).

In the present study, it was shown that exclusive cytoplasmic protein VCL plays a role in the cell nucleus in specific cell types such as meiocytes. Its depletion affects prophase I progression, resulting in prophase I arrest and decreased fertility in males. Moreover, it associates with proteins from the UPS, which has only recently been found to be essential to meiotic progression, during which the place and time when the proteins are degraded is critical. However, the real involvement of VCL in the synaptonemal complex assembly, centromeric stabilization, and UPS action in these processes must still be explored. 

## 4. Discussion

### 4.1. VCL Might Be a New Kinetochore Component

Centromeric pairing is not exclusive to the mouse model. In fact, it has been described in a variety of organisms and specifically termed “centromere coupling” in yeast, for example [72]. It has been shown that SYCP1, SYCP3, and synapses of pericentromeric chromatin persist in later stages and are important factors for proper meiotic chromosomal segregation [11,16,73]. Centromere cohesion is protected from separase by the presence of Shugoshin-2 (Sgo2), thus allowing sister chromatids to remain tightly attached as they are moved towards the spindle poles during anaphase I [74,75]. Maintaining this connection between sisters throughout the entirety of anaphase and telophase of meiosis I is essential since prematurely individualized sister chromatids run the risk of being mis-segregated in meiosis II. Other factors involved in this early stage of G2/MI transition remain unknown. We showed that VCL appeared in the centromeric region of WT primary spermatocytes. After its depletion, centromeres appeared synapsed in pachytene, but not in diplotene. The centromere of the bivalents split and separated. Moreover, at a closer view, it was possible to recognize the centromeres of two sister chromatids, although they were stabilized by the SCP3 lateral element yet significant as two couples of defined foci (super-resolution microscopy). In addition, a mitotic spindle (a self-assembling macromolecular machine responsible for the faithful segregation of chromosomes during cell division) was assembled according to the “Search & Capture” principle. It means that the dynamic microtubules explore space in search of kinetochores, while the latter capture microtubules and thus connect chromosomes to the spindle. We noted that the size and shape of kinetochores in addition to their distribution in space were crucial at the onset of spindle assembly. We hypothesize that VCL is a new component of the meiotic chromosome kinetochore. In co-immunoprecipitated proteins, we find one partner which may support this idea. RBBP4 (see set of nuclear interacting proteins, Supplementary Figure S5) and RBBP7 are proteins of the NuRD (Nucleosome Remodeling Deacetylase) complex, which participates in the assembly of centromeric histone variant CENPA and is a spindle assembly factor. It allows the formation of a Ran-GTP gradient emanating from condensed chromosomes to catalyze microtubule polymerization and spindle assembly [76]. We showed that in VCL^cKO^ spermatocytes, the spindle was non-standard and mostly collapsed. Our hypothesis and observations support the idea that VCL might be a novel component of meiotic kinetochore, and our indirectly obtained data agree with the previously shown close relationship between kinetochore integrity and spindle assembly. Nevertheless, more experimentation is needed to prove this hypothesis and to discover VCL’s direct action in meiocytes.

### 4.2. Vinculin Associates with UPS and Probably Plays a Dual Role

The UPS’s involvement in meiotic events was recently described in mammalian spermatogenesis by Rao et al. [17]. These studies point to the important role of the UPS in the epistatic regulation of meiotic proteins for successful prophase I progression. Using structured illumination microscopy (SIM), they observed localization of the majority of ubiquitin foci at the axes of the synaptonemal complex (SC) between homologs. Proteasomes were recruited along the axes during zygonema and persisted throughout pachynema and diplonema when chromosomes de-synapsed. Proteasome foci are largely axis-associated, but less frequent in the SC central region [23,24,26]. Meiosis-specific proteasome association with chromosomes was shown as a 20S proteasome subunit signal overlapping with the SYCP3-po chromosome axes. This pattern is abundant during all meiotic stages except leptonema, in contrast to non-meiotic testicular cells where staining is absent [17,21]. Modification and further degradation of proteins in the nucleus between homologously juxtaposed chromosomes and crossing over are essential for proper homologous pairing, formation of synapsis, and chiasma formation. Without a proteasome function, homologs fail to pair and remain associated with non-homologous chromosomes. We found that VCL interacts with components of UPS machinery (Figure 8). We co-localized it with the proteasome complex, but colocalization was not as frequent as we expected. In pachynema, places of VCL-PSMB1 co-localization were evident and usually surrounding fully synapsed bivalents. Fragments without full cohesion of bivalents were evident (apparent as SCP3-positive sister chromatids), and colocalization of VCL and PSMB1 was not detected. We have no explanation for this phenomenon, but the interaction between these two proteins is possibly dynamic and transient. We also found a variable pattern in PSMB1 staining, seen as a scattered signal in the nuclear area (Figure 9A) and closely attached to the pachynema bivalents (Figure 9B). This variable proteasome pattern has not yet been reported, and we suggest it is highly dynamic and that only mid-pachytene meiotic nucleus possess proteasome complexes dominantly aligned along the fully synapsed bivalents. Nevertheless, in spermatocytes without VCL, even in the mid-pachytene stage, we observed no proteasome complexes aligned along the SCs, but interestingly, generally fewer signals were also evident in the nucleoplasm. This observation might suggest not only colocalization, but also co-distribution of VCL and proteasome subunits. We emphasize that VCL appears in two conformations—opened and closed—where the closed conformation is an auto-inhibitory state (head-to-tail self-association). The structure of activated vinculin remains unknown, and only fragments of the molecule are known in its interacting state [73,77]. Originally, Stec and Stec [78] published the unique crystal structure, showing VCL to form bundles and even super-bundles. This is reminiscent of a bundle of pencils tied in the middle. Such an architecture clearly suggests that the dominant mode of mobility for the entire molecule is a twisting motion, leading to partial unrolling and unfurling of the entire molecule. Moreover, they suggest that this unique attribute provides a highly dynamic but also extremely stable hub for interacting proteins (such as Arp2/3, actinin, talin, and others), forming large protein complexes. It is also important to highlight that no data are currently available for proteasome complex assembly in meiocytes since it is such an enormously large structure. From this point of view, VCL appears to be a strong candidate for putting the pieces of the proteasomal jigsaw together in its essential role during mammalian meiosis.

## Data Availability

Data are contained within the article or Supplementary Materials.

## Supplementary Materials

The following supporting information can be downloaded at: https://www.mdpi.com/article/10.3390/cells11132013/s1, Figure S1: epididymal sperm count and formation of acrosome in WT and VCLcKO males; Figure S2: measuring the distance between two CREST foci in WT and VCL^cKO^ pachytene and diplotene spermatocytes; Figure S3: FACS profile of enriched diplotene spermatocytes in VCL^cKO^ males; Figure S4: metaphase I chromosome spreads showing disorganized cohesion on the chromosomal arms in VCL^cKO^ males; Figure S5: VCL co-immunoprecipitated proteins form spermatocytes’ cytoplasmic and nuclear lysate; Figure S6: SUMO immunodetection and quantification in WT and VCL^cKO^ pachytene spermatocytes.
