# Supplementary material for: Focal Adhesion Protein Vinculin Is Required for Proper Meiotic Progression during Mouse Spermatogenesis
Source: Cells. 2022 Jun 23;11(13):2013. doi: 10.3390/cells11132013 (PMC9265697; doi:10.3390/cells11132013)
Supplement: Supplementary file 1 [file cells-11-02013-s001.zip › cells-1773689-supplementary.pdf]

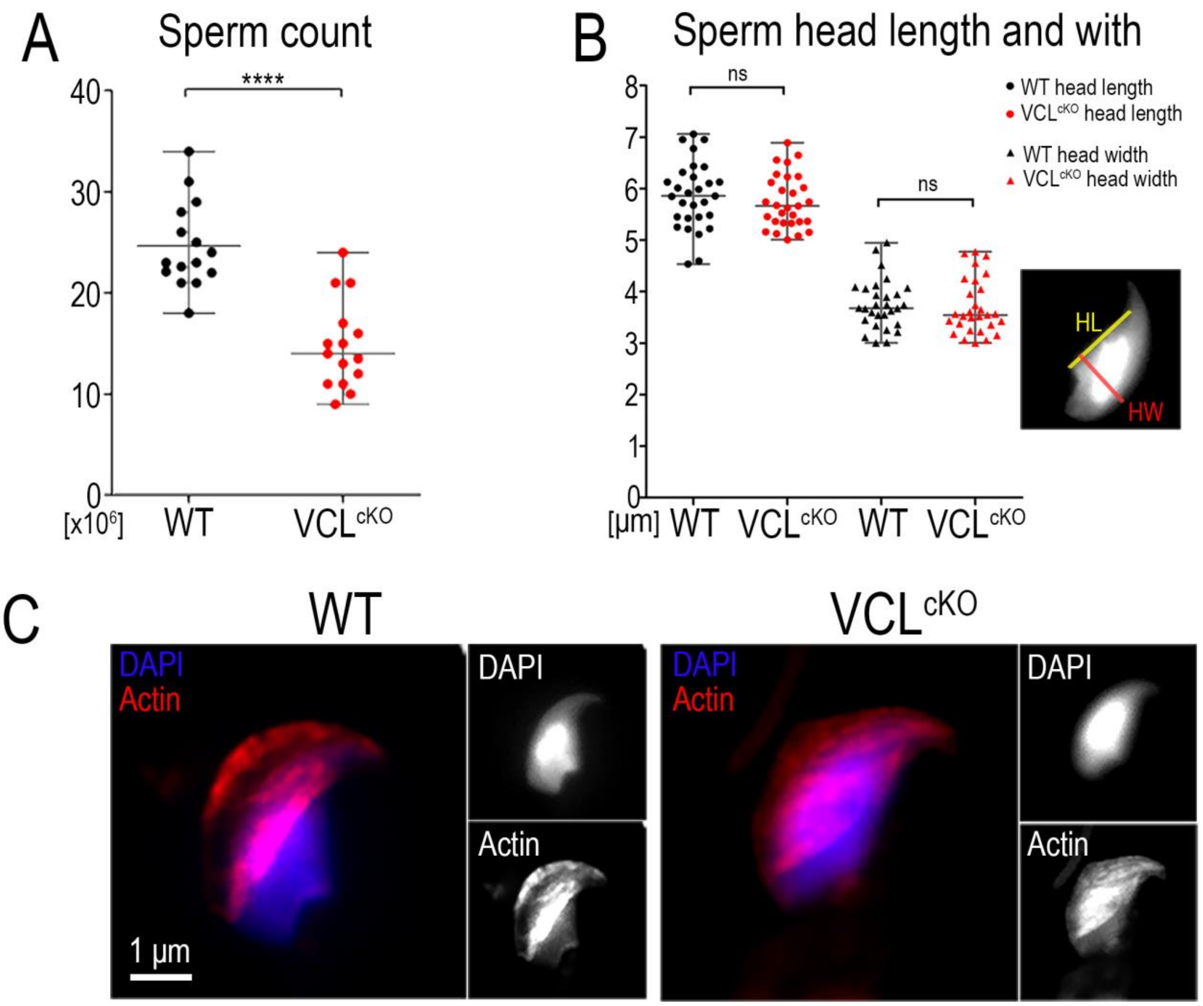

Supplementary figure S1. (A) Epididymis sperm count of WT and VCL^{cKO} shows significant decrease ~ 50% of sperm ammount in VCL depleted males. Statistical evaluation by unpaired t test **** correspond to $p < 0.0001$. **(B)** Quantitative determination of sperm head morphology, i.e. head length and width, measured as indicated in the detail on the right. HL = Head length; HW = head width. Statistical evaluation by unpaired t test (head length p = 0.2411 (ns), head width p = 0.5960 (ns). **(C)** Localization of phalloidin stained polymeric actin as an indicator of possible defects in formation of acrosome, a major determinant of abrogated fertility. VCLcKO sperm possess the same acrosome formation as in WT control males.

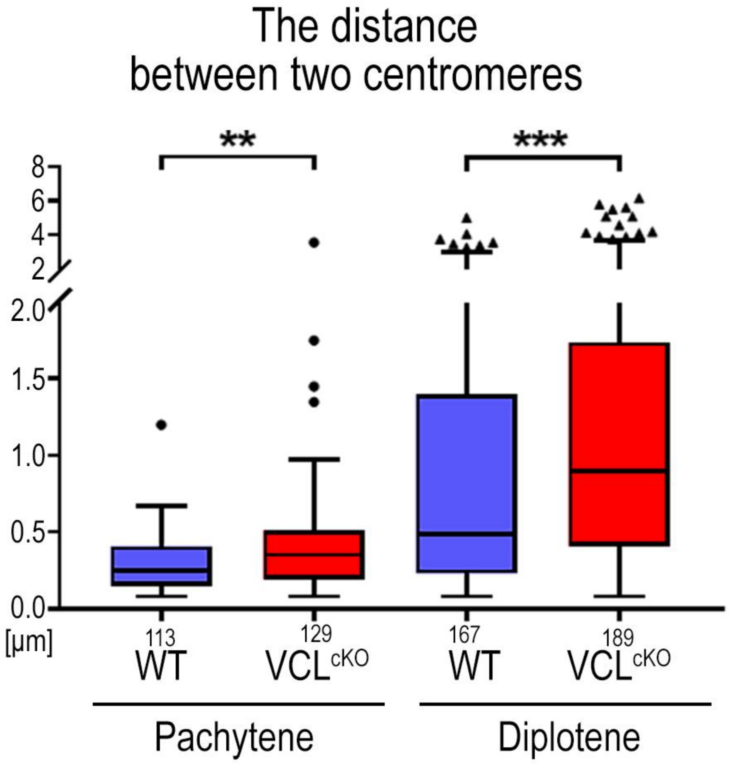

Supplementary figure S2. The distance measurement between the two CREST foci. The de-synapsed centromeres of homologous kinetochore are significantly more distant VCL^{cKO} pachytene and diplotene nuclei than in WT control males. The number of synapses scored by measurement of the distance between two CREST foci are indicated below the columns. Statistical evaluation by unpaired t test ** correspond to p= 0.0023 and *** represents p= 0.00012.

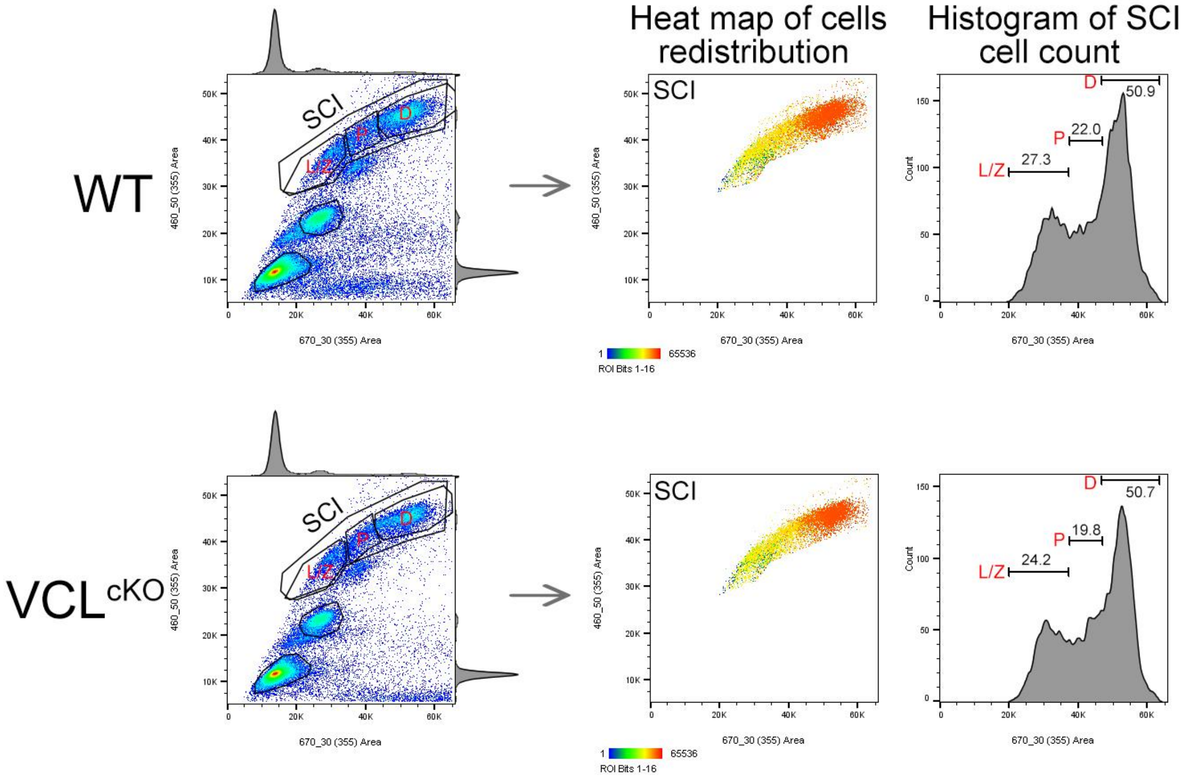

Supplementary figure S3. Single cell suspension from WT and VCL^{cKO} testes stained with Hoechst-33342 and visualized within two emission spectra. The gating strategy was to identify whole SCI population for specification of cell count comparing to the rest of the testicular cells. SCI population was then divided into three populations (in scythe part of the plot - low, mid and high) containing the prophase I spermatocytes – i.e. leptotene/zygotene mixed population (L/Z), pachytene (P) and diplotene (D) spermatocytes. SCI population was then visualized with frequency heat plot, where accumulation of cell in the diplotene area is apparent in VCL^{cKO} sample. This observation together with decreased number of cells in L/Z and P (histogram on the right side) of VLC^{cKO} males points to the metaphase I arrest of VCL depleted meiocytes.

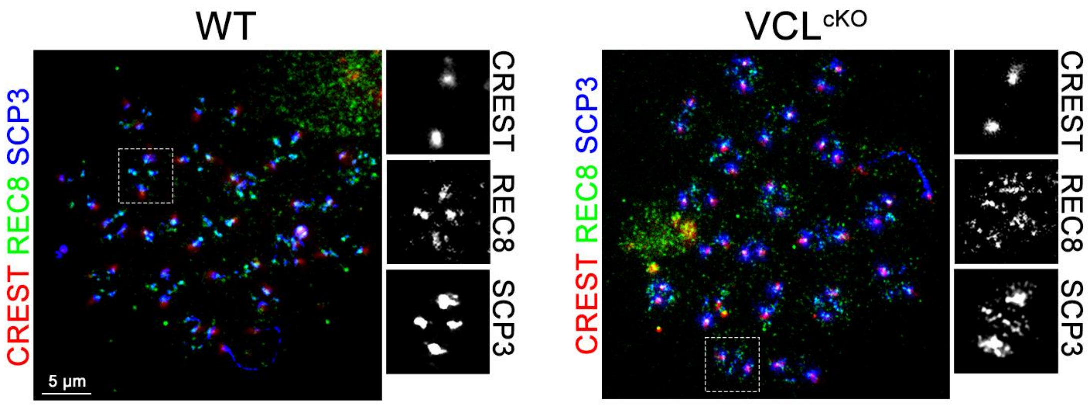

Supplementary figure S4. Metaphase I chromosome spreads contains disorganized cohesion on the chromosomal arms when VCL is depleted. Metaphase I spreads were stained with CREST (centromeres, red), REC8 (cohesion, green) and SCP3 (axial synaptonemal protein, blue). In WT control expected pattern of cohesins is apparent – REC8 localizes to the short and long arm of the chromosome bivalent, colocalizing with SCP3. Place of chiasma (middle of the cross) is the place of cross over and does not contain any axial element. Centromeres are at the termini of long arm crucial for attachment of the bivalent to the microtubular spindle. By contrast, in VCL depleted spermatocytes in metaphase I, REC8 appears in the dotty disorganized pattern, only partially colocalizing with SCP3. Moreover, SCP3 localizes not only at the termini of the chromosomal arms, but also in the chiasma.

Actins, ABPs and ARPs		Protein abundance
Actc1	Actin, alpha cardiac muscle 1	1.0-1.2
Actg1	Actin, cytoplasmic 2	1.5-1.6
Acta1	Actin, Alpha skeletal muscle	1.0-1.2
Capza1	F-actin-capping protein subunit alpha-1	1.0-1.2
Capza2	F-actin-capping protein subunit alpha-2	1.5-1.6
Capzb	F-actin-capping protein subunit beta	1.5-1.6
Actn4	Alpha-actinin-4	1.5-1.6
Actr3	Actin-related protein 3	1.0-1.2
Arpc1b	Actin-related protein 2/3 complex subunit 1B	1.3-1.4
Arpc2	Actin-related protein 2/3 complex subunit 2	1.0-1.2
Arpc4	Actin-related protein 2/3 complex subunit 4	1.3-1.4
Flna	Filamin-A	1.0-1.2
Tuba1b	Tubulin alpha-1B chain	2.0<
Tuba4a	Tubulin alpha-4A chain	1.5-1.6
Tubb4b	Tubulin beta-4B chain	1.5-1.6
Vim	Vimentin	1.0-1.2
Twf	Twinfilin-1	1.0-1.2

New associated cytoplasmic VCL partners		Protein abundance
Ano10	Anoctamin-10	1.3-1.4
Cfl1	Cofilin-1	1.0-1.2
Cttn	Src substrate cortactin	1.0-1.2
Dync1li2	Cytoplasmic dynein 1 light intermediate chain 2	1.0-1.2
Dynlrb2	Dynein light chain roadblock-type 2	1.0-1.2
Tpt1	Translationally-controlled tumor protein	2.0<
Tex101	Testis-expressed sequence 101 protein	1.3-1.4
Izumo4	Izumo sperm-egg fusion protein 4	1.7-1.8

Cell nucleus		Protein abundance
Nup93	Nuclear pore complex protein Nup93	1.0-1.2
Rbbp4	Histone-binding protein RBBP4	1.5-1.6
Cse1l	Exportin-2	1.0-1.2
Kpna4	Importin subunit alpha-3	1.3-1.4
Kpnb1	Importin subunit beta-1	1.5-1.6

Meiosis		Protein abundance
Piwil1	Piwi-like protein 1	1.0-1.2
Piwil2	Piwi-like protein 2	1.3-1.4
Tsn	Translin	1.0-1.2
Ruvbl1	RuvB-like 1	1.7-1.8
Ruvbl2	RuvB-like 2	2.0<

Protein abundance: <1.0; 1.0-1.2; 1.3-1.4; 1.5-1.6; 1.7-1.8; 1.9-2.0; 2.0<

Supplementary figure S5. Co-immunoprecipitation of VCL from spermatocytes' cytoplasmic and nuclear lysate revealed new possible VCL partners.

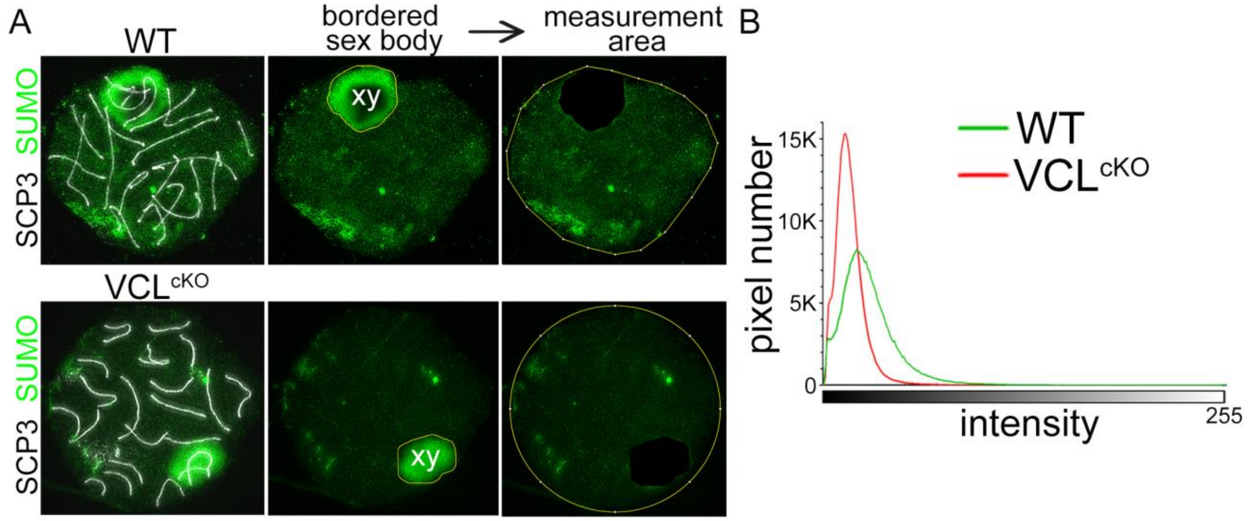

Supplementary figure S6. (A) SUMO immunolocalization and signal quantification. In WT VCL^{cKO} was apparent strong signal in sex body (depicted xy) in pachytene spermatocytes. To get the information about the reduction of diffused nucleoplasm SUMO signal, the area of the sex body was excluded (middle panel). The intensities were evaluated only from ROI with excluded xy (yellow ROI, right panel). **(B)** Quantification of reduced signal (graph) by color histogram (Fiji ImageJ).
